# Factors Affecting the Success of Ovum Pick-Up, In Vitro Production and Cryopreservation of Embryos in Cattle

**DOI:** 10.3390/ani15030344

**Published:** 2025-01-25

**Authors:** Farzaneh Salek, Alysha Guest, Chinju Johnson, John P. Kastelic, Jacob Thundathil

**Affiliations:** Faculty of Veterinary Medicine, University of Calgary, Calgary, AB T2N 4N1, Canadachinju.johnson@ucalgary.ca (C.J.); jpkastel@ucalgary.ca (J.P.K.)

**Keywords:** OPU, bovine embryos, embryo cryopreservation, L-carnitine, Hippo signaling

## Abstract

Increasing global demand for animal proteins warrants improved productivity by genetic selection of superior cattle and faster dissemination of genetics. Availability of more progeny for genomic selection should maximize chances of identifying animals with desirable traits and increase selection pressure. OPU and IVP of embryos using these oocytes will substantially increase calves produced compared to conventional embryo transfer (ET). The OPU-IVP technology not only supports genetic improvement but also contributes to reducing environmental impacts of livestock production systems by improving efficiency and optimizing resources, aligning with the Sustainable Development Goals of the United Nations. However, there are several factors influencing the success of OPU-IVP. This review is focused on these factors and the impacts of in vitro culture conditions on the lipid content of embryos and potential role of L-carnitine, a lipolytic agent, on developmental competence of IVP embryos. The documented effects of L-carnitine and current knowledge regarding regulation of the Hippo signaling pathway suggest that supplementation of embryo culture media with L-carnitine will increase post-thaw survival of IVP embryos and their subsequent developmental competence by regulating lipid metabolism, production of reactive oxygen species, and Hippo signaling. Therefore, this review highlights current advancements in the field of OPU-IVP and potential areas for refining culture conditions to yield developmentally competent embryos that survive cryopreservation procedures.

## Simple Summary

Repeated ultrasound-guided oocyte aspiration (ovum pick-up; OPU) and in vitro production (IVP) of embryos are fundamental techniques in cattle breeding that enhance genetic progress by increasing the number of embryos produced. Hormonal stimulation improves the number and quality of oocytes retrieved, resulting in higher blastocyst development rates. IVP techniques focus on improving embryo development in vitro, with advancements in culture conditions enhancing overall embryo quality and cryosurvival. High lipid content in bovine IVP embryos can affect post-thaw survival of embryos. Strategies like lipid reduction and supplementation with lipolytic agents (e.g., L-carnitine) are being explored to improve cryotolerance of embryos. The Hippo signaling pathway which regulates cell growth and development, has also been involved in embryo development, offering a potential target for improving embryo competence and survival during cryopreservation. This review is focused on factors affecting the success of OPU-IVP and improving the cryosurvival of resulting embryos.

## 1. Introduction

In recent decades, assisted reproductive technologies (ARTs) have gained substantial importance in humans and animals. Since a burgeoning global population requires increased food production, there is a need to improve cattle productivity [1]. Existing and emerging reproductive technologies are fundamental to maximizing progeny born to superior cows, facilitating selection for improved productivity and dissemination of superior genetics globally [2]. Improvements in livestock productivity can boost food production for a growing world population.

In vitro production (IVP) of embryos and conventional embryo transfer (ET) are commonly used for propagating offspring from superior beef and dairy cattle. However, reproductive efficiency can be increased several-fold by repeated ultrasound-guided ovum pick-up and IVP of embryos (OPU-IVP), which could boost livestock productivity and environmental sustainability due to maintenance of fewer cattle and less greenhouse gas emissions [3]. For instance, livestock production contributes to climate change through methane, carbon dioxide, and nitrous oxide emissions; therefore, selective breeding and genetic advancements to improve productivity per animal are effective strategies to reduce greenhouse gas emissions per unit of food produced [4,5]. Indeed, the generation interval of cattle can be reduced, as OPU can be performed before puberty or up to 3 months of pregnancy and oocytes can be fertilized with sperm from more than one bull or with sexed semen (with no reduction in fertility) [6,7]. Sex-sorted sperm can be used to produce female embryos from young heifers with high genetic value [8,9]. Furthermore, IVP technology provides valuable insights into human infertility and supports conservation of endangered and commercially important animals. Moreover, OPU-IVP is a viable option for cattle with reduced fertility due to reproductive tract pathology, impaired sperm transport, or terminal diseases [10].

During the last decade, there has been a substantial global increase in IVP of embryos and their cryopreservation [10]. Although worldwide in vivo bovine embryo production has largely stabilized, IVP of bovine embryos was 31.5% higher in 2021 versus 2020 (International Embryo Technology Society data) [11], with 34.5% and 37.0% increases in North and South America, respectively. Canada consistently ranks within the top 10 countries globally in exporting beef and other cattle products. Canada has ~11.5 million cattle (9.5 million beef and 2 million dairy), with >40% of Canada’s beef herd in western Canada [12,13]. The overall objective of this review is to discuss current advancements in the field of OPU-IVP and potential areas for refining culture conditions to yield developmentally competent embryos that survive cryopreservation procedures. Specifically, the objectives of this review are to: (1) discuss the factors influencing the success of OPU-IVP; (2) analyze the effects of cryopreservation on embryos and how the lipid composition of embryos impacts their cryosurvival; and (3) evaluate recent improvements in in vitro culture conditions to optimize the lipid composition of IVP embryos, particularly the role of L-carnitine supplementation and the underlying molecular mechanisms of its beneficial effects. This review benefits OPU-IVP teams and researchers seeking to improve the efficiency of OPU-IVP procedures. Such advancements will increase the reproductive efficiency of superior cattle, addressing global demands for increased food production while minimizing the environmental impact of cattle production systems.

## 2. Methodology Applied for the Search and Selection of Articles

The literature reviewed in the current paper was sourced from comprehensive searches conducted in the following databases: SCOPUS, Web of Science, PubMed, Science Direct, Google Scholar. The literature search covered publications up to 2024 to include both foundational and current research. Keywords were used for the bibliographic search in various combinations to ensure broad coverage of relevant studies were: “bovine oocytes”, “ovum pick-up”, “in vitro production”, “OPU-IVP”, “factors affecting OPU”, “hormonal stimulation”, “IVP embryos”, “embryo cryopreservation”, “lipid metabolism”, “L-carnitine”, and “Hippo signaling pathway”. Articles were included based on their relevance to the topic, methodological rigor, and focus on bovine assisted reproductive technologies. Exclusion criteria were as follows: studies not available in English, not peer-reviewed, or not specifically addressing the key focus areas of this review.

## 3. Factors Affecting Success of OPU-IVP

### 3.1. Donor-Dependent Factors

Success of OPU-IVP can be influenced by various factors, including oocyte quality, donor parity, lactational status, nutritional status, donor age, and management practices [6,14,15,16]. Parturition and post-partum periods are critical phases characterized by physiological changes including calving, uterine involution, and lactation. These changes, along with metabolic and hormonal alterations, immune system suppression, and elevated risk of inflammation, affect overall reproductive health and performance in cattle [17,18]. For instance, lactating dairy cows generally have lower oocyte quality and fertilization rates compared to non-lactating cows. This reduction is mostly attributed to lactational metabolic challenges, which compromise early blastocyst development and elevate the risk of metabolic and infectious diseases during the postpartum period [19,20]. In addition, high milk production promotes steroid metabolism, reducing blood progesterone and estradiol concentrations and decreasing estrus expression [21]. The success of OPU can be influenced by blood concentrations of estrogen and progesterone, which play important roles in regulating the reproductive cycle. These hormones support follicular development during ovarian stimulation. However, in high-producing lactating dairy cows, metabolic challenges often lead to hormonal imbalances, characterized by lower progesterone and estrogen concentrations. Such imbalances can impair follicular dynamics, resulting in development of larger ovarian follicles that compromise oocyte quality and reduce the number of viable and high-quality oocytes for retrieval during OPU, ultimately affecting subsequent embryonic development [22,23,24]. Serbetci et al. (2024) reported that postpartum cows with subclinical metabolic or inflammatory disease have reduced oocyte quality due to a suboptimal follicular environment [17]. Another study evaluated the developmental potential of oocytes collected from dairy cows at various postpartum stages and reported that despite metabolic changes associated with calving and lactation, oocyte developmental competence was not compromised [25]. Additionally, numerous studies highlighted the complex relationship between nutrition and reproduction, revealing that dietary intake and composition, feeding levels, and body condition score affect fertility, particularly through altered ovarian function [26,27]. Dantas et al. emphasized the importance of complexed trace minerals (zinc, copper, and manganese, and cobalt glucoheptonate) in enhancing OPU-IVP outcomes by improving oocyte quality and embryo production in lactating beef cows compared to those receiving inorganic trace minerals [28]. Furthermore, short-term supplementation of a high-energy diet in Japanese Black cows elevated insulin concentration, the number of ovarian follicles, and recovered oocytes. However, this diet reduced oocyte quality, resulting in lower cleavage rates without improving blastocyst production compared to a maintenance diet. This study highlighted the importance of addressing the adverse effects of excessive dietary supplementation on developmental competence of oocytes [29]. Therefore, nutrient deficiencies and negative energy balance can impair oocyte developmental competence and embryo production, whereas overfeeding can also negatively affect oocyte competence [27,30]. Meeting nutritional requirements is crucial for promoting reproductive success in cattle.

Oocyte quality and in vitro embryo development can vary between breeds and subspecies [31]. British or Continental taurus cattle (*Bos taurus*) and indicus cattle (Zebu, *Bos indicus*) are subspecies characterized by distinct physiological differences, including heat tolerance level, age at puberty, gestation length, ovarian follicular dynamics, ovarian follicle count and diameter, estrus length, and circulating hormone concentrations [32,33,34,35]. For instance, the success rate of IVP is usually higher for oocytes derived from *Bos indicus* compared to oocytes from *Bos taurus*, both beef and dairy [36,37]. This success of indicus cattle is attributed to their larger reservoir of antral follicles, which enhances oocyte recovery [38]. Consequently, IVP outcomes tend to be more efficient in indicus than in taurus due to more recovered oocytes and blastocysts produced per OPU session [36,37,39]. Another study reported that *Bos taurus* had lower plasma Anti mullerian hormone (AMH) concentrations and a smaller population of ovarian antral follicles compared to *Bos indicus* when managed under identical conditions [35]. Therefore, a deep understanding of physiological differences is essential for developing targeted reproductive management protocols. These insights enhance practices such as artificial insemination, ovarian superstimulation, and embryo production to optimize reproductive efficiency in cattle.

Based on a recent study by Thundathil et al. [40], although purebred Angus and Hereford cows differed in their OPU-IVP responses, in general, IVP outcomes were higher in cows than those from crossbred heifers (Table 1). Perhaps this reflects the impact of genetic backgrounds of the cows on the OPU-IVP outcomes.

Several studies have investigated impacts of donor age on oocyte retrieval and blastocyst development [41,42]. Younger animals have a larger reserve of ovarian follicles, resulting in more oocytes harvested during OPU session compared to adult donors. However, calves and heifers have been linked to reduced blastocyst yields and pregnancy rates due to lower developmental competence of their oocytes compared to those from mature cows [43,44,45]. In that regard, oocytes recovered from adult cows (>4 years) had considerably higher developmental competence than those from heifers (<30 months), with 46.5% vs. 33.4% viable blastocysts, respectively, with blastocysts not significantly different among heifers aged 12–18, 19–24, or 25–30 months [46]. In that study, postpartum cows had smaller ovarian follicle reserves and similar developmental competence of embryos, likely due to metabolic stress. Another study investigated IVP using oocytes obtained from cows and heifers of a herd infected with bovine spongiform encephalopathy; more cow versus heifer oocytes reached the blastocyst stage for freezing and subsequent transfer (21.8% vs. 15.6%, respectively) [47].

### 3.2. Superstimulation-Dependent Factors

Increasing the number and quality of oocytes during aspiration is an important goal that can be achieved through superstimulation. Among various hormones, follicle stimulating hormone (FSH) is known to improve OPU outcomes [48,49]. Synchronizing follicular wave emergence and using FSH for superstimulation enhances oocyte quality and promotes blastocyst development [8]. Repeated superstimulation followed by OPU-IVP increases the number and size of viable oocytes and embryos obtained from superior donors by enhancing rates of cleavage and morula and blastocyst development [50,51]. This approach also reduces the generation interval, accelerating genetic improvement. Thus, there is increasing adoption of OPU-IVP after superstimulation due to its greater efficiency in embryo production [52]. However, excessive or prolonged ovarian superstimulation may reduce ovarian responsiveness over time. This decline can negatively affect the quality and developmental competence of oocytes, potentially impacting embryo production efficiency. This highlights the need for optimized protocols to balance yield with long-term reproductive health [53,54,55]. In *Bos indicus* cattle, for instance, FSH superstimulation had no positive effect on IVP outcomes, and non-superstimulated cows had higher rates of hatched blastocysts; perhaps there are breed-specific variations in response to ovarian superstimulation [56].

Regarding oocyte competency, superstimulation of young donors can significantly boost their embryo developmental potential to nearly match adult cows [44,57]. Currin et al. evaluated the effects of age and duration of gonadotropin stimulation (long-term, short-term, or no stimulation) on oocyte quality and embryo development in Holstein calves aged 2–6 months. There was an age-related increase in oocyte developmental competence, with older calves achieving higher blastocyst rates compared to their younger counterparts. Moreover, long-term stimulation enhanced the proportion of large follicles, improved oocyte quality, and significantly increased blastocyst rates (36.7%) compared to short-term (18.3%) or no stimulation (16.7%), highlighting the efficacy of extended gonadotropin treatment [58]. In another study, exogenous hormonal stimulation in Holstein calves at 2 to 3 months and 4 to 5 months significantly increased the number of follicles [59]. In contrast, in a study exploring the effects of FSH stimulation on developmental potential of oocytes aspirated from 3-month-old calves versus adult cows, the cleavage rate was similar, but the embryo production rate was significantly higher in cows (>20%) than in unstimulated and stimulated calves (9% and 11%, respectively). Lower developmental competence in younger donors (e.g., 3 months-old), even with hormonal stimulation, may be related to a suboptimal follicular microenvironment before puberty [45]. Oocytes collected from prepubertal calves exhibit limited developmental competence due to the immaturity of the hypothalamic-pituitary-ovarian (HPO) axis, which impairs their ability to undergo complete cytoplasmic and nuclear maturation compared to those from adult cows [60]. Consequently, an effective hormonal stimulation protocol may promote a better follicular microenvironment and support follicle growth, increase follicular diameters, and enhance oocyte competence prior to OPU or laparoscopic ovum pick-up (LOPU) [61,62,63].

### 3.3. Procedure-Dependent Factors

In cattle, inducing ovarian stimulation and subsequently allowing a gonadotropin-free interval is commonly referred to as “coasting”. This procedure can improve oocyte quality and blastocyst yields [44,50]. Numerous studies modified superstimulation protocols, including various coasting intervals, to allow oocytes complete cytoplasmic maturation along with nuclear maturation. Coasting for 48 h was reported to increase the number of 5–10 mm follicles compared to 33 h of coasting [50]; FSH superstimulation with a 48-h coasting period and giving LH 6 h prior OPU optimized follicular development and blastocyst yields (80.4%). Coasting also improved the developmental potential of oocytes and embryo production in Holstein *(Bos taurus)* cows [64]. In Italian Mediterranean buffalo, administration of FSH (40 mg, six times) along with a shorter coasting period (28–32 h) optimized ovarian superstimulation, including number of aspirated follicles (especially medium and large), higher oocyte quality, and greater blastocyst yields, whereas more prolonged coasting (64–68 h) negatively affected oocyte quality and embryo production in buffalo [65]. Several studies have linked higher blastocyst production rates to increased numbers of follicles after FSH stimulation [66,67]. Oocyte competence increases during follicular growth with molecular changes, as oocyte gene expression varies depending on follicle size; exogenous FSH may improve this process by promoting medium-sized follicles that accumulate key regulatory genes [67,68,69].

To evaluate impacts of prolonged superstimulation and multiple OPU-IVP cycles on blastocyst development and donor health in cattle, repeated superstimulation followed by OPU-IVP was performed for >1 year on six cross-bred beef heifers (12–14 months old) and three purebred beef cows [40]. In brief, a CIDR was inserted intravaginally (Day 0), with transvaginal aspiration of dominant follicles ≥ 10 mm on Day 2. FSH injections were given 12 h apart beginning on Day 4 (AM) and ending on Day 6 PM (total of six treatments). Transvaginal ultrasound-guided oocyte aspiration was conducted after 38 to 40 h of coasting (interval between last FSH and oocyte collection). On Day 8, ovarian follicles were aspirated using an ultrasound-guided transvaginal approach. The COCs were immediately recovered and used for IVP procedure [40].

**Figure 1:**
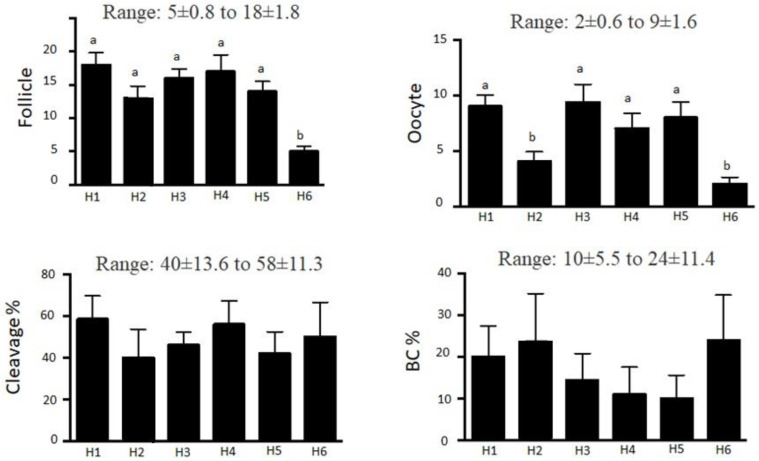
Mean ± SEM effects of repeated OPU-IVP cycles over 1 year (data compiled from nine trials) in crossbred heifers (*n* = 6). BC = Blastocyst production. ^a,b^ Within an end point, heifers without a common superscript differed (*p* < 0.05).

In crossbred heifers, repeated OPU-IVP trials did not affect IVP outcomes, with production of viable embryos (Table 2). However, the number of follicles, collected oocytes, and cleavage and blastocyst rates varied substantially among individual animals (Figure 1). Although OPU is a valuable ART, there are concerns regarding adverse impacts on animal welfare, as the procedure is considered invasive and may cause stress or discomfort, especially with repeated sessions [70,71]. It has been suggested that repeated OPU may increase stress and decrease reproductive herd performance, though it did not appear to affect reproductive ability [72]. Repeated egg recovery over one year (nine OPU-IVP sessions) and prolonged superstimulation followed by OPU-IVP did not compromise reproductive health of heifers, as these donors were subsequently used as embryo recipients and all that became pregnant delivered a viable calf.

These results suggest that multiple OPU-IVP cycles can be effectively performed for a prolonged interval to maximize embryo production from elite donors without compromising reproductive health. However, repeated OPU may cause ovarian scar tissue formation, adhesions, hemorrhage, or infection [73]. It has been demonstrated [40] that all cattle tolerated multiple sessions of OPU-IVP without any undesirable effects on reproductive or general health, consistent with previous reports [70,74].

## 4. In Vitro Culture Conditions and OPU-IVP Outcomes

Oocytes recovered by OPU can be matured in the laboratory (in vitro maturation; IVM), fertilized (in vitro fertilization; IVF), and grown (in vitro culture; IVC) until they become blastocysts, which are then freshly transferred to recipient cows (foster mothers) or cryopreserved for future use [10]. Improvement in each stage of IVP and cryopreservation can improve embryo quality, rates of pregnancy, and numbers of live births. Conditions under which an oocyte matures in vitro significantly differ from those matured in vivo [75]. Alteration of maturation conditions can affect the developmental competence of oocytes, reflected by blastocyst yield. Oocytes and early-stage embryos are highly sensitive to changes in diverse exogenous factors, including temperature, osmolarity, oxygen, pH, amino acids, and lipids. The culture environment exposes oocytes to a variety of cellular stresses that contribute to the loss of their developmental competence. During in vivo development, an embryo is exposed to variable fluid compositions, nutrition, and gas atmospheres as it travels from the oviduct to the uterus. Numerous studies have measured oxygen concentrations in the uterine tube and uterus of various mammals; oxygen concentration in the uterine tube is typically 5% to 8%, whereas uterine concentrations were even lower [76,77,78]. These studies suggested that low oxygen concentrations were crucial for embryonic development, as increased concentrations can generate reactive oxygen species (ROS), leading to cellular damage [79]. The developmental potential and stress-related responses of mouse embryos cultured under varying oxygen concentrations (2 and 5%) were compared against in vivo-derived blastocysts. There were higher development rates in blastocysts developed in vivo and under 5% oxygen than those cultured under 2%, whereas stress-response and apoptosis genes were elevated in embryos cultured under 2% oxygen [80]. Increasing the oxygen concentration to 20% markedly reduced both blastocyst yield and post-vitrification survival rates, highlighting the detrimental effects of increased oxygen on embryo viability [81]. These studies emphasized the importance of oxygen regulation in embryo culture systems for improving developmental outcomes in ART. Accordingly, the success of IVC depends on how closely in vivo conditions are replicated.

There are various embryo culture systems, including static culture and sequential culture. Static culture uses a single formulation of culture media, allowing an embryo to select required components [82]. The premise of a sequential culture system is that during development, preimplantation embryos have diverse requirements and are exposed to a variety of secreted factors during migration to the uterus, as discussed above; therefore, media and its components are changed based on embryo stage and stage-specific embryonic demands [83]. Both methods have advantages and disadvantages. Sequential culture mimics in vivo conditions but may cause more oxidative stress by changing media and increased handling. In contrast, static media allow decreased disturbance of pH and temperature, reducing ROS production and allowing embryos to benefit from secreted growth factors, although metabolic waste products and ROS can be deleterious [10]. Reduced efficiency of IVP embryos and their poor cryosurvival compared to in vivo-derived embryos [84] can be linked to suboptimal culture conditions, which can disrupt oocyte and embryo metabolism.

## 5. Impacts of Cryopreservation on IVP Embryos

Cryopreservation enables prolonged preservation of oocytes and embryos; the latter can be thawed and transferred to recipients, facilitating large-scale use of IVP embryos [10]. In particular, cryopreservation aims to retain cell structure and functionality and essentially suspend metabolism and enzymatic activity. The two main methods for cryopreservation are slow freezing and vitrification; both have been employed for oocyte and embryo cryopreservation in several species, with mostly satisfactory results. For cryopreservation of in vitro bovine embryos, the slow freezing method involves cooling embryos from room temperature to –35 °C at a controlled rate of cooling (0.5 °C/min), followed by indefinite storage in liquid nitrogen at −196 °C [85]. Unfortunately, the viability of frozen-thawed IVP embryos remains low, which may be due to altered metabolism and perturbation of the lipid profile. Freezing tolerance is highly dependent on these lipids; embryos with altered lipid composition and content are more sensitive to cryoinjury. Furthermore, during cryopreservation, lipid phase transition alters conformation states of lipid molecules from a fluid phase to a solid-like phase that can cause cryodamage [86].

**Figure 2:**
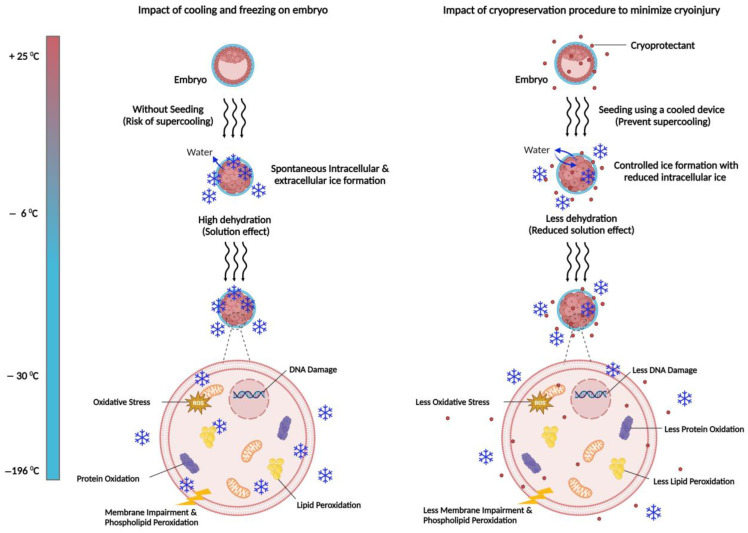
Cryopreservation can disrupt the embryo viability by cryoinjuries and altering cellular function. The major causes of cryodamage are intracellular ice formation (IIF) and reactive oxygen species (ROS) production which lead to oxidative stress, triggering lipid peroxidation, protein oxidation, and impairing the membrane integrity. In addition, dehydration during freezing causes embryo contraction (as illustrated, the embryo shrinks, and the blastocoel is no longer visible). An ideal cytoprotective agent mitigates these negative effects of cooling and freezing on cells. The penetrating cryoprotectants, hygroscopic in nature, bind with extracellular and intracellular water and thus reduce ice growth, regulate osmotic pressure, and reduce ROS production (by converting them into harmless substances to minimize ROS-induced damage). Therefore, the addition of cryoprotectant(s) in medium prior to freezing is essential for minimizing cryopreservation-related injuries but must be carefully managed to avoid toxicity and interference with cellular structures. Seeding with a liquid nitrogen-cooled device prevents supercooling and controls ice formation, as cells with higher solute concentration avoid intracellular ice formation, unlike rapid ice crystal formation in unseeded freezing. Created with BioRender.com.

Cryodamage typically occurs during lipid phase transition and lipid peroxidation events that occur during freezing and thawing [87]. During freezing, oocytes and embryos are exposed to cryoprotectants like dimethyl sulfoxide (DMSO), glycerol, and ethylene glycol (EG) for lowering the freezing point to prevent cryoinjuries during freezing and thawing [88]. Due to their lipophilic nature, these agents pass through the cellular membranes to manage osmotic pressure and prevent intracellular ice crystal formation by cell dehydration before freezing [89]. One of the main challenges in cryopreservation is supercooling, where water is cooled below its freezing point without forming ice, remaining in a liquid state. This can lead to spontaneous ice formation in a supercooled solution, increasing the risk of damaging the embryo by allowing ice to form inside the cells. To mitigate these risks, it is crucial to induce extracellular ice formation near the freezing point, either mechanically or with ice-nucleating agents [90]. Seeding (inducing ice nucleation) during embryo cryopreservation is essential for preventing supercooling and ensuring a successful cryopreservation procedure. By manually inducing ice formation at temperatures between –5 °C and –7 °C, seeding facilitates gradual cellular dehydration and controlled ice propagation, minimizing the risk of intracellular ice formation. During cryopreservation procedure, seeding ensures safer ice formation and better embryo protection [91]. In vitro culture conditions and cryopreservation can increase reactive oxygen species (ROS) in IVP embryos, leading to oxidative stress and negatively impacting cryosurvival [92]. The majority of IVP embryos display features linked to lower quality, including vacuoles in trophoblastic cells, a decreased number of mitochondria and microvilli, diminished intercellular junctions, variations in gene expression, and changes in lipid metabolism [93,94,95]. The accumulation of ROS in cells is controlled by three key enzymes. Superoxide dismutase (SOD) reduces superoxide radicals (O_2_^•−^) by converting it to hydrogen peroxide (H_2_O_2_), which is then mitigated through two enzymatic pathways. Catalase (CAT) enzyme converts two molecules of H_2_O_2_ into water (H_2_O) and oxygen (O_2_). The second pathway is mediated by glutathione peroxidase (GPx), which uses glutathione (GSH) as an electron donor to reduce H_2_O_2_ to H_2_O. Together, these enzymatic antioxidants minimize ROS accumulation, protecting cells from oxidative damage [96,97]. Non-enzymatic antioxidants work synergistically with enzymatic antioxidants to defend cells from free radical damage. These antioxidants can be endogenous or sourced exogenously through diet or dietary supplements [98]. Various studies on antioxidant supplements like cysteine, selenium, hypotaurine, melatonin, resveratrol, and L-carnitine reported their effectiveness in improving oocyte maturation, fertilization, embryo development, and cryotolerance [96,99,100,101,102]. However, critical challenges in cryopreservation, including cell toxicity from ROS, DNA damage, mitochondrial dysfunction, and lipid peroxidation (Figure 2), still need to be addressed to improve the effectiveness of cryopreservation.

We compared the developmental competence of in vivo- and in vitro-derived embryos when they were transferred fresh or frozen-thawed to recipient cows. There were no statistically significant differences in pregnancy rates between in vivo- and in vitro-derived embryos when they were transferred fresh (63.0% versus 51.7%, respectively). However, pregnancy rates of in vivo-produced frozen embryos (53.9%) were significantly greater than that of IVP frozen embryos (33.3%), indicating the need for further refinement of embryo culture and freezing procedures. Therefore, producing embryos that tolerate cryopreservation is an important challenge of OPU-IVP [84]. Strategies to increase cryotolerance of IVP embryos include culture under low O_2_ to minimize oxidative stress, use of antioxidants or apoptosis inhibitors [103,104], or use of substances to accelerate lipid metabolism or prevent lipid accumulation [105]. Although lipids are essential for energy metabolism, their high content in IVP embryos make these cells more susceptible to cryopreservation-induced damage [106].

## 6. Lipid Composition and Metabolism of In Vivo Versus In Vitro Embryos

Lipids are essential biomolecules produced de novo in the endoplasmic reticulum or derived from the in vitro culture environment [107]. Fatty acids (FA) are important components of lipids that form structural components of membranes and are stored as neutral lipids inside lipid droplets (LDs), eventually being metabolized to provide substrates for energy production and signaling. Energy production through lipid β-oxidation has a vital role in supporting energy demand during oocyte maturation, and blastocysts expansion, and hatching [108,109]. Many FAs are esterified to glycerol (catalyzed by diacylglycerol acyltransferase; DGAT) and stored as neutral triacylglycerols (TAG), the major lipid class in the cytoplasm of mammalian cells. However, the main lipids in cell membranes are phospholipids (PLs).

Degradation of lipid droplets occurs via lipolysis of TAGs to fatty acyl-CoA by lipases, making FAs available for metabolism [110]. In addition, there is a regulatory mechanism mediated by Perilipin Adipophilin Tail-interacting Protein of 47 kD (PAT) family in oocytes and embryos to control lipid storage. The PAT family consists of Perilipin (PLIN) 1, 2, and 3, and can restrict or facilitate the activity of lipases in lipid droplets. The PAT family directs interactions between lipases and lipid droplets. For example, PLIN2 colocalized with lipid droplets and was associated with lipid accumulation in bovine oocytes and embryos [111,112].

Alterations in lipid metabolism have been related to developmental competence and cryotolerance of oocytes and embryos [113,114]. Lipid composition in IVP embryos differs from in vivo due to in vitro culture conditions and media composition [115], compromising cryosurvival. Lipid accumulation is explained by absorption of serum lipoproteins, synthesis of triglycerides in the presence of serum, and reduced β-oxidation of lipids in mitochondria [115]. Lipids, especially polyunsaturated fatty acids (PUFAs), are highly prone to oxidation and are primary targets for lipid peroxidation. Thus, intracellular fatty acids excess and accumulation (e.g., diacylglycerols) are linked to high lipid peroxidation, leading to oxidant/antioxidant balance, disrupted β-oxidation, ER stress, and cellular dysfunction [116,117]. Lipid peroxidation may be related to fetal bovine serum (FBS) supplementation due to its high lipid content and the presence of PUFAs in FBS [118,119]. Perhaps, then, it is not surprising that embryos deriving from oocytes matured in serum-supplemented media are reported to have reduced quality and that the addition of an antioxidant like vitamin E elevated the rate of embryo development [120]. Lipid peroxidation can block embryo development in mice [121]. Thus, it is necessary to optimize the culture conditions to reduce cytoplasmic accumulation of lipids.

Phospholipid composition is crucial to the physical and chemical properties (e.g., fluidity and permeability) of the cell membrane. Membrane phospholipids, including phosphatidylcholine, phosphatidylinositol, phosphatidylserine, phosphatidylglycerol, and phosphatidylethanolamine, are structural units of functional membranes in mammalian cells and the most abundant lipids in the eukaryotic membrane [115]. Thus, any changes in FA composition of cell membrane will have major impacts on cell function through altered metabolism and signaling mechanisms [115,122]. Alteration of physical and chemical characteristics of membranes can also affect cryotolerance. For example, low temperatures during cryopreservation of oocytes and embryos often lead to chilling damage [123,124]. Therefore, higher cryosensitivity of IVP embryos may be partly due to altered membrane phospholipid composition. Techniques that alter the lipid content of oocytes and embryos can increase embryonic survival [95]. Lipid depletion in oocytes and embryos by micromanipulation has been used to enhance cryotolerance [125,126], but also has important disadvantages. Mechanical lipid removal from oocytes/embryos increases pathogen transmission due to breaching the zona pellucida [127]. Furthermore, this technique is also particularly labor intensive.

Non-invasive methods to reduce intracellular lipid accumulation and modify plasma membrane composition to mimic those characteristics of in vivo embryos will be key to the success of oocytes and embryo culture [128]. Some of these lipolytic agents include epinephrine, norepinephrine, isoproterenol, forskolin, and carnitine. These compounds have the potential to promote lipolysis through various mechanism and signaling pathways compared to traditional methods [115]. Panyaboriban et al. (2018) demonstrated that forskolin, through its lipolytic action, effectively reduced intracellular lipid content in bovine and buffalo embryos, enhancing cryotolerance and post-thaw developmental competence, particularly in bovine embryos. This highlights its potential as a chemical delipidation agent to mitigate lipid-related cryodamage during cryopreservation [129]. Additionally, extracellular vesicles (EVs) have gained attention for their ability to mimic in vivo conditions owing to their rich molecular content. EVs play a pivotal role in intercellular signaling and regulating diverse physiological processes. A study demonstrated that EVs from oviductal and uterine fluid enhance bovine embryo quality by reducing lipid content and influencing lipid metabolism-related genes and lipase activation [130]. Following this study, it was reported that miRNAs within EVs (oviduct and uterus) may regulate bovine embryo lipid metabolism and development, although their specific roles remain unclear [131]. The precise molecular pathways and regulatory mechanisms through which lipolytic agents influence lipid regulation remain unclear. Beyond lipid breakdown, these agents may impact other critical metabolic and cellular processes by modulating key regulatory networks and signaling pathways. This underscores the critical need for a deeper understanding of the role of these agents and their interactions with key metabolic pathways and regulatory networks.

Culture media during IVP can be supplemented with FBS and bovine serum albumin (BSA), which has varying lipid contents. Although FBS is commonly added to culture media to provide essential nutrients, growth factors, and other components that support growth and development, oocytes matured in media supplemented with FBS accumulated more lipid than those in media containing BSA [118]. Although withdrawal of FBS can reduce lipid accumulation and improve cryotolerance, it often compromises embryo development [46,132]. Freezing tolerance is highly dependent on these lipids, and embryos with altered lipid composition and content are more sensitive to cryoinjury. Cryodamage typically occurs during lipid phase transition and lipid peroxidation events [87], as described below. Improving the OPU-IVP procedure and cryopreservation should increase embryo quality and live births.

## 7. Carnitine

One of the most effective non-invasive techniques to reduce intracellular lipid content is the addition of lipolytic agents that can cleave or modify lipids, rendering them unharmful [113]. Carnitine is a non-proteinogenic amino acid that is an important part of various energy metabolism pathways due to its role as a lipid metabolism regulator. L-carnitine is a biologically active form of carnitine naturally present in animals and derived from lysine and methionine [133,134,135]. Carnitine is present in human follicular fluid and has been associated with improved human fertility [75,136,137]. Although neither oocytes nor cumulus cells can biosynthesize L-carnitine from precursor amino acids [137], it may be possible to improve in vitro culture conditions by supplementing media with L-carnitine during oocyte and embryo growth, thereby enhancing lipid β-oxidation and antioxidant properties.

L-carnitine serves as a cofactor for transporting long-chain fatty acids (derived from TAG hydrolysis) from the cytosol to the mitochondria [138]. However, in addition to ATP production, long-chain FA metabolism generates ROS that can inflict oxidative damage and negatively impact cryopreservation efficiency. Crucially, L-carnitine is a potent antioxidant that scavenges ROS, thereby improving blastocyst formation and post-thaw survival of cryopreserved IVP embryos [139]. L-carnitine has potential to boost mitochondrial function and promote β-oxidation in bovine and murine oocytes, respectively [140,141]. L-carnitine treatment enhanced nuclear maturation, increased numbers of active mitochondria in porcine oocytes, reduced intracellular lipids, and enhanced preimplantation development in bovine blastocysts [108]. In addition to its role in fatty acid β-oxidation (FAO), carnitine also regulates membrane fluidity by adjusting phospholipid content in the membrane. In fact, carnitine is integral to regulatory turnover of membrane phospholipids [142,143,144]. Therefore, L-carnitine supplementation promotes cell membrane stability through its involvement in acetylation of phospholipids and its amphiphilic function within the cell membrane [136,145].

Furthermore, L-carnitine can prevent reduction of ATP and control total phospholipid concentrations in cells [142,146]. For example, decreases in phosphatidylcholine, phosphatidylinositol, and phosphatidylethanolamine content were effectively prevented with L-carnitine [147]. In addition, L-carnitine protected the stability of buffalo oocyte membranes by maintaining physiological concentrations of desirable phospholipids (phosphatidylcholine, phosphatidylinositol, and phosphatidylethanolamine) [146]. Therefore, adding L-carnitine to culture media not only reduces cytoplasmic lipid content through mitochondrial FA modulation but also stabilizes membrane phospholipid composition, thereby increasing membrane fluidity and survival after cryopreservation. In a study on the impacts of L-carnitine (1.5 mM) with varying FBS concentrations (2.5%, 5%, 7.5% and 10%) on embryo development and cryosurvival, supplementation of embryo culture media with L-carnitine and 2.5% or 5% FBS resulted in a higher rate of embryo production with enhanced post-thaw cryosurvival in comparison to higher levels of FBS [148].

## 8. Hippo Signaling Pathway

**Figure 3:**
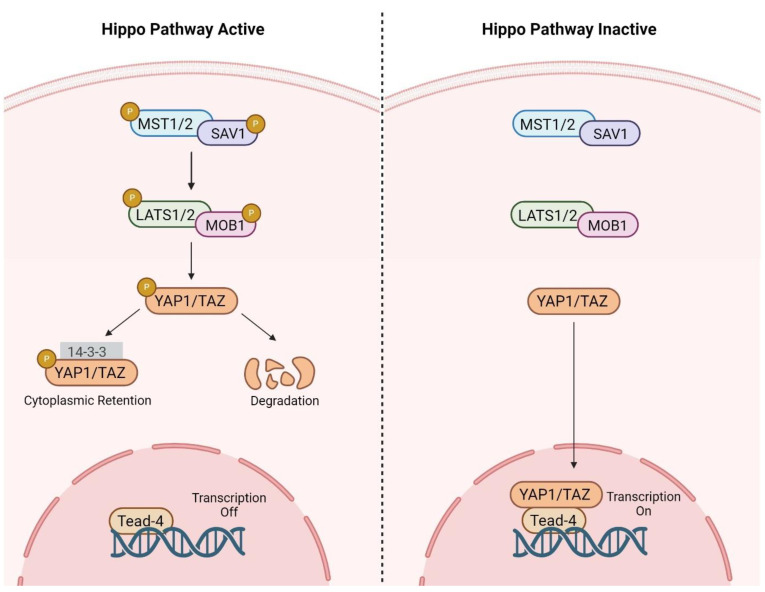
Hippo signaling pathway components in mammals. The Hippo signaling pathway has a crucial role in regulating cell proliferation, apoptosis, and organ size. When the pathway is active, upstream signals trigger phosphorylation (P) of MST1/MST2 kinases. SAV1 functions as a scaffold protein, forming a complex with MST1/2 kinases. This interaction facilitates phosphorylation and activation of LATS1/2 kinases. MOB1A/B function as co-factors for LATS1/2 kinases; they form a complex with LATS1/2, enhancing phosphorylation and activation of YAP/TAZ proteins, which leads to YAP/TAZ retention in the cytoplasm or their degradation. As a result, YAP/TAZ cannot enter the nucleus or promote gene expression related to cell growth. However, when the pathway is inactive, YAP/TAZ are unphosphorylated, translocate to the nucleus, and activate TEAD, promoting expression of genes necessary for cellular growth and migration. MST1/2: mammalian ste20-like kinase; LATS1/2: large tumor suppressor kinase; SAV1: scaffold protein Salvador; YAP: Yes-associated protein; TAZ: transcriptional co-activator with PDZ-binding motif; TEAD: TEA domain family member; and P: phosphorylation. Created with BioRender.com.

Considering the role of lipids in regulating various cellular processes, including energy production, membrane stability, and signaling pathways, a deeper understanding of these processes will serve as a future avenue for optimizing in vitro production of developmentally competent embryos. Numerous signaling pathways, including wingless-type MMTV integration site family (Wnt), Notch, mitogen-activated protein kinases (MAPKs), and Hippo, have important roles in folliculogenesis and embryonic development through regulation of proliferation, apoptosis, and differentiation of embryonic cells like inner cell mass (ICM) and trophectoderm (TE) [149,150]. The Hippo pathway is composed of a serine/threonine kinase cascade involving upstream regulators, core cascade components, and downstream effectors [149]. During preimplantation embryogenesis, this pathway is triggered by cell-cell contact involving apical polarity proteins like angiomotin (AMOT), partitioning defective 6 homolog (PARD6), and neurofibromin 2 (NF2). These proteins facilitate cell contact and polarity, which in turn activates phosphorylation, and subsequently activate the core cascade components of the pathway [151] (Figure 3). The Hippo signaling pathway consists of mammalian sterile 20-like 1/2 (MST1/2) and large tumor suppressor kinase 1/2 (LATS1/2) as core components. MST1/2 interacts with the scaffolding proteins Salvador homolog 1 (SAV1) and neurofibromatosis type 2 (NF2/Merlin), as well as with LATS1/2; the latter interacts with MOB kinase activators 1A and B (MOB1A and B) [152]. Activation of MST1/2 and LATS1/2 leads to phosphorylation of downstream effectors of the Hippo pathway. Yes-associated protein (YAP) and transcriptional coactivator with PDZ binding motif (TAZ) are two important downstream effectors of the Hippo pathway and their activation (through dephosphorylation) and inhibition (through phosphorylation) status regulate expression of downstream genes [153].

When the Hippo signaling pathway is activated, YAP/TAZ are inhibited via phosphorylation and remain in the cytosol. In contrast, when the Hippo signaling pathway is inactive, YAP/TAZ are activated (dephosphorylated) and consequently translocate into the nucleus. Activation of YAP/TAZ stimulates gene expression through TEA domain transcription factor (TEAD). Within the nucleus, TEAD regulates transcription of developmentally important genes such as *caudal type homeobox 2* (CDX2, a marker of trophectoderm), *sex determining region Y-box 2* (SOX2, a marker of embryonic inner cell mass), and *octamer-binding transcription factor 4* (OCT4, a marker of pluripotent cells) [151,154].

Although the Hippo pathway is conserved in cattle and mice, there may be species differences in the localization of Hippo signaling components in blastocysts [149,155]. Regulation of the Hippo pathway can be achieved through various effectors such as cell-cell contact, extracellular signals, cellular polarity, metabolic conditions, and mechanotransduction [156]. Based on recent studies, membrane phospholipids play important roles in various intracellular signaling pathways. Since L-carnitine modulates lipid content and composition, it is likely that it has a role in regulation of the Hippo signaling pathway. Phospholipids such as phosphatidic acid, lysophospholipids, sphingosine1-phosphophate, and phosphoinositol can regulate the Hippo pathway through dephosphorylation and subsequent activation of YAP/TAZ [151]. For instance, a bioactive phospholipid lysophosphatidic acid (LPA) inhibits Hippo pathway kinases LATS1 and LATS2 via Ga12/13-coupled receptors and increases YAP concentrations in bovine embryos by preventing its phosphorylation and degradation.

Crucially, LPA accelerated the onset of blastocyst formation. When embryos were treated with LPA, expressions of YAP, TAZ and TEAD4 were increased [157]. Another lipid, phosphatidic acid (PA), also regulates the Hippo pathway by acting as a second messenger to decrease YAP phosphorylation. When the amount of membrane-associated PA is low, the enzyme phospholipase D can catalyze phosphatidylcholine hydrolysis to produce PA. According to Han et al. (2018), PA physically interacts with LATS and an upstream component of the pathway NF2. PA disrupts formation of LATS-MOB1 complexes and LATS membrane translocation and activation by NF2, ultimately leading to inhibition of Hippo signaling. Interestingly, treatment with PA activates YAP even under conditions that typically promote Hippo activation [158].

Several studies have highlighted the role of AMP-activated protein kinase (AMPK) as a suppressor of YAP/TAZ activity, linking cellular energy dynamics to regulation of Hippo signaling effectors [159,160]. AMPK plays a pivotal role in cellular energy homeostasis by regulating glucose and lipid metabolism. Increased AMP/ATP ratios during low energy states activate AMPK, which regulates cellular lipid metabolism by phosphorylating acetyl-CoA carboxylase (ACC). This phosphorylation reduces malonyl-CoA levels, a molecule that normally inhibits carnitine palmitoyltransferase 1 (CPT1). By decreasing malonyl-CoA concentrations, AMPK removes CPT1 inhibition and facilitates fatty acid β-oxidation. Under energy stress, AMPK activation can directly phosphorylate and inactivate YAP and result in YAP nuclear exclusion [161,162]. The role of L-carnitine through enhancing fatty acid β-oxidation on AMPK signaling also has been demonstrated [163,164,165]. Therefore, L-carnitine may exert a regulatory effect on YAP through AMPK signaling.

Additionally, lipid metabolism may affect Hippo signaling by modulating YAP and TAZ activity through the mevalonate pathway [166] that converts acetyl-coenzyme A into lipid precursors (like cholesterol, isoprenoid) and other metabolites. Geranylgeranyl pyrophosphate, an intermediate product of the mevalonate pathway, can activate Rho GTPases that inhibit phosphorylation of YAP and TAZ, which results in YAP activation and translocation into the cell nucleus [167,168,169,170]. Inhibition of the mevalonate pathway reduces nuclear localization and activity of YAP and TAZ, ultimately suppressing cell growth [167].

Considering the impacts of lipids on regulating the Hippo signaling pathway and the key role of L-carnitine in facilitating fatty acid oxidation, and stabilizing phospholipids [136,171], L-carnitine potentially exerts regulatory effects on the Hippo signaling pathway through its involvement in lipid metabolism and homeostasis. Therefore, supplementation of embryo culture media with L-carnitine may enhance post-thaw survival of IVP embryos and their subsequent developmental competence by regulating lipid metabolism, ROS production, and Hippo signaling.

## 9. Conclusions

Improving the efficiency of OPU-IVP procedures without compromising the wellbeing of donor animals will improve animal productivity to help meet global demands for animal proteins. Furthermore, adopting OPU-IVP technologies can contribute to reducing the environmental impacts of cattle production by improving efficiency and sustainability—an increasingly critical goal in modern livestock systems. Although many improvements in embryo culture conditions have been made and several commercial media are available, further research is required to develop evidence-based approaches for improving developmental competence and post-thaw survival of embryos. Although L-carnitine has been extensively used in embryo culture media, further research is required to elucidate its mechanisms of actions. Based on the role of phospholipids on the Hippo pathway and the documented role of L-carnitine on regulation of lipid metabolism, we inferred that the beneficial effects of L-carnitine on IVP are mediated through a mechanism involving the Hippo signaling pathway. Future studies could investigate the potential role of L-carnitine in regulating the Hippo signaling pathway, focusing on its interactions with lipid metabolism, phospholipid stabilization, and ROS management. Investigations should aim to determine whether L-carnitine affects Hippo pathway components or its upstream regulators and how these interactions might influence embryonic development and competence. This investigation could provide valuable insights into the possible regulatory effects of L-carnitine on the Hippo pathway and its broader implications for improving embryonic outcomes.

## Figures and Tables

**Table 1:** Mean ± SEM effects of repeated OPU-IVP in purebred beef cows (*n* = 3).

Cow (No. Trials)	No. Follicles	No. Oocytes	Cleavage (%)	Blastocyst Rate (%)
A (2)	56.0 ± 1.0 ^a^	17.5 ± 3.5 ^a^	54.5 ± 16.5	20.0 ± 1 ^a^
B (4)	24.7 ± 5.6 ^b^	9.5 ± 2.3 ^ab^	70 ± 15.9	52.5 ± 18.3 ^ab^
C (4)	9.0 ± 1.2 ^c^	4 ± 0.8 ^b^	89 ± 6.2	78 ± 7.7 ^b^

^a–c^ Within a column, means without a common superscript differed (*p* < 0.05).

**Table 2:** Mean ± SEM effects of repeated OPU-IVP trials on ovarian responses, oocyte collection, and embryonic development in crossbred beef heifers (*n* = 6).

Trial	No. Follicles	No. Oocytes	Cleavage (%)	Blastocyst Rate (%)
1	14 ± 2.8	6 ± 1.7	66 ± 15	31 ± 14.2
2	14 ± 3.5	6 ± 1.1	67 ± 5.5	20 ± 13.3
3	19 ± 2.4	11 ± 2.1	68 ± 7.9	20 ± 7.1
4	16 ± 2.0	9 ± 1.1	56 ± 8.2	23 ± 11.5
5	14 ± 2.9	5 ± 2.1	42 ± 17	39 ± 13.1
6	11 ± 2.1	5 ± 1.1	34 ± 16.2	18 ± 10.2
7	13 ± 2.1	7 ± 1.8	27 ± 13	5.5 ± 3.7
8	12 ± 2.4	6 ± 2.5	44 ± 12.1	15 ± 7.1
9	12 ± 2.4	6 ± 1.2	33 ± 17.1	0

## Data Availability

Data are contained within the article.

## References

[1] Daly J., Smith H., McGrice H.A., Kind K.L., Van Wettere W.H.E.J. (2020). Towards Improving the Outcomes of Assisted Reproductive Technologies of Cattle and Sheep, with Particular Focus on Recipient Management. Animals.

[2] Rexroad C., Vallet J., Matukumalli L.K., Reecy J., Bickhart D., Blackburn H., Boggess M., Cheng H., Clutter A., Cockett N. (2019). Genome to Phenome: Improving Animal Health, Production, and Well-Being—A New Usda Blueprint for Animal Genome Research 2018–2027. Front. Genet..

[3] Davis T.C., White R.R. (2020). Breeding Animals to Feed People: The Many Roles of Animal Reproduction in Ensuring Global Food Security. Theriogenology.

[4] Hansen P.J. (2023). Some Challenges and Unrealized Opportunities toward Widespread Use of the in Vitro-Produced Embryo in Cattle Production. Animal.

[5] Balmford A., Amano T., Bartlett H., Chadwick D., Collins A., Edwards D., Field R., Garnsworthy P., Green R., Smith P. (2018). The Environmental Costs and Benefits of High-Yield Farming. Nat. Sustain..

[6] Humblot P., Le Bourhis D., Fritz S., Colleau J.J., Gonzalez C., Guyader Joly C., Malafosse A., Heyman Y., Amigues Y., Tissier M. (2010). Reproductive Technologies and Genomic Selection in Cattle. Vet. Med. Int..

[7] Bó G.A., Cedeño A., Mapletoft R.J. (2019). Strategies to Increment in Vivo and in Vitro Embryo Production and Transfer in Cattle. Anim. Reprod..

[8] Demetrio D.G.B., Benedetti E., Demetrio C.G.B., Fonseca J., Oliveira M., Magalhaes A., Santos R.M.D. (2020). How Can We Improve Embryo Production and Pregnancy Outcomes of Holstein Embryos Produced in Vitro? (12 Years of Practical Results at a California Dairy Farm). Anim. Reprod..

[9] Soares J.G., Martins C.M., Carvalho N.A.T., Nicacio A.C., Abreu-Silva A.L., Campos Filho E.P., Torres Júnior J.R.S., Sá Filho M.F., Baruselli P.S. (2011). Timing of Insemination Using Sex-Sorted Sperm in Embryo Production with Bos Indicus and Bos Taurus Superovulated Donors. Anim. Reprod. Sci..

[10] Ferré L.B., Kjelland M.E., Strøbech L.B., Hyttel P., Mermillod P., Ross P.J. (2020). Review: Recent Advances in Bovine in Vitro Embryo Production: Reproductive Biotechnology History and Methods. Animal.

[11] Viana J.H. (2022). 2021 Statistics of Embryo Production and Transfer in Domestic Farm Animals. Embryo Technol. Newsl..

[12] Greenwood P.L. (2021). Review: An Overview of Beef Production from Pasture and Feedlot Globally, as Demand for Beef and the Need for Sustainable Practices Increase. Animal.

[13] Cordeiro M.R.C., Mengistu G.F., Pogue S.J., Legesse G., Gunte K.E., Taylor A.M., Ominski K.H., Beauchemin K.A., McGeough E.J., Faramarzi M. (2022). Assessing Feed Security for Beef Production within Livestock-Intensive Regions. Agric. Syst..

[14] Ferré L.B., Alvarez-Gallardo H., Romo S., Fresno C., Stroud T., Stroud B., Lindsey B., Kjelland M.E. (2023). Transvaginal Ultrasound-guided Oocyte Retrieval in Cattle: State-of-the-art and Its Impact on the in Vitro Fertilization Embryo Production Outcome. Reprod. Domest. Anim..

[15] Albarrán-Portillo B., Pollott G.E. (2013). The Relationship between Fertility and Lactation Characteristics in Holstein Cows on United Kingdom Commercial Dairy Farms. J. Dairy Sci..

[16] Bezdíček J., Nesvadbová A., Makarevich A., Kubovičová E. (2020). Relationship between the Animal Body Condition and Reproduction: The Biotechnological Aspects. Arch. Anim. Breed..

[17] Serbetci I., González-Grajales L.A., Herrera C., Ibanescu I., Tekin M., Melean M., Magata F., Malama E., Bollwein H., Scarlet D. (2024). Impact of Negative Energy Balance and Postpartum Diseases during the Transition Period on Oocyte Quality and Embryonic Development in Dairy Cows. Front. Vet. Sci..

[18] Pascottini O.B., Leroy J.L.M.R., Opsomer G. (2020). Metabolic Stress in the Transition Period of Dairy Cows: Focusing on the Prepartum Period. Animals.

[19] Leroy J.L.M.R., Rizos D., Sturmey R., Bossaert P., Gutierrez-Adan A., Van Hoeck V., Valckx S., Bols P.E.J. (2012). Intrafollicular Conditions as a Major Link between Maternal Metabolism and Oocyte Quality: A Focus on Dairy Cow Fertility. Reprod. Fertil. Dev..

[20] Ribeiro E.S., Gomes G., Greco L.F., Cerri R.L.A., Vieira-Neto A., Monteiro P.L.J., Lima F.S., Bisinotto R.S., Thatcher W.W., Santos J.E.P. (2016). Carryover Effect of Postpartum Inflammatory Diseases on Developmental Biology and Fertility in Lactating Dairy Cows. J. Dairy Sci..

[21] Lopez H., Caraviello D.Z., Satter L.D., Fricke P.M., Wiltbank M.C. (2005). Relationship between Level of Milk Production and Multiple Ovulations in Lactating Dairy Cows. J. Dairy Sci..

[22] Wiltbank M., Lopez H., Sartori R., Sangsritavong S., Gümen A. (2006). Changes in Reproductive Physiology of Lactating Dairy Cows Due to Elevated Steroid Metabolism. Theriogenology.

[23] Missio D., Fritzen A., Cupper Vieira C., Germano Ferst J., Farias Fiorenza M., Guedes De Andrade L., Martins De Menezes B., Tomazele Rovani M., Gazieira Gasperin B., Dias Gonçalves P.B. (2022). Increased β-Hydroxybutyrate (BHBA) Concentration Affect Follicular Growth in Cattle. Anim. Reprod. Sci..

[24] Argov N., Arav A., Sklan D. (2004). Number of Oocytes Obtained from Cows by OPU in Early, but Not Late Lactation Increased with Plasma Insulin and Estradiol Concentrations and Expression of mRNA of the FSH Receptor in Granulosa Cells. Theriogenology.

[25] Matoba S., O’Hara L., Carter F., Kelly A.K., Fair T., Rizos D., Lonergan P. (2012). The Association between Metabolic Parameters and Oocyte Quality Early and Late Postpartum in Holstein Dairy Cows. J. Dairy Sci..

[26] Ponter A.A., Guyader-Joly C., Nuttinck F., Grimard B., Humblot P. (2012). Oocyte and Embryo Production and Quality after OPU-IVF in Dairy Heifers given Diets Varying in Their n-6/n-3 Fatty Acid Ratio. Theriogenology.

[27] Velazquez M.A. (2023). Nutritional Strategies to Promote Bovine Oocyte Quality for In Vitro Embryo Production: Do They Really Work?. Vet. Sci..

[28] Dantas F.G., Reese S.T., Filho R.V.O., Carvalho R.S., Franco G.A., Abbott C.R., Payton R.R., Edwards J.L., Russell J.R., Smith J.K. (2019). Effect of Complexed Trace Minerals on Cumulus-Oocyte Complex Recovery and in Vitro Embryo Production in Beef Cattle1,2. J. Anim. Sci..

[29] Tomita K., Ishii T., Endo N., Tanaka T. (2023). Effects of Short-Term Dietary Supplementation on the Number of Ovarian Follicles, Quantity and Quality of Oocytes, and *in Vitro* Embryo Production in Japanese Black Cows. J. Reprod. Dev..

[30] Reynolds L.P., Vonnahme K.A. (2017). Livestock as Models for Developmental Programming. Anim. Front..

[31] Abraham M.C., Ruete A., Brandt Y.C.B. (2010). 260 Breed Influences Outcome of in Vitro Production of Embryos in Cattle. Reprod. Fertil. Dev..

[32] Monteiro C.A.S., Saraiva H.F.R.D.A., Leal G.R., Camargo A.J.D.R., Serapião R.V., Ferreira A.M.R., Rodrigues A.L.R., Nogueira L.A.G., Oliveira C.S. (2017). Breed Composition Does Not Influence the Performance of Holstein-Gyr Crossbred as Oocyte Donors for OPU/IVP. Anim. Reprod..

[33] Sartori R., Monteiro P.L.J., Wiltbank M.C. (2016). Endocrine and Metabolic Differences between Bos Taurus and Bos Indicus Cows Andimplications for Reproductive Management. Anim. Reprod..

[34] Cooke R.F., Cardoso R.C., Cerri R.L.A., Lamb G.C., Pohler K.G., Riley D.G., Vasconcelos J.L.M. (2020). Cattle Adapted to Tropical and Subtropical Environments: Genetic and Reproductive Considerations. J. Anim. Sci..

[35] Baldrighi J., Sá Filho M., Batista E., Lopes R., Visintin J., Baruselli P., Assumpção M. (2014). Anti-Mullerian Hormone Concentration and Antral Ovarian Follicle Population in Murrah Heifers Compared to Holstein and Gyr Kept Under the Same Management. Reprod. Domest. Anim..

[36] Pontes J.H.F., Silva K.C.F., Basso A.C., Rigo A.G., Ferreira C.R., Santos G.M.G., Sanches B.V., Porcionato J.P.F., Vieira P.H.S., Faifer F.S. (2010). Large-Scale in Vitro Embryo Production and Pregnancy Rates from Bos Taurus, Bos Indicus, and Indicus-Taurus Dairy Cows Using Sexed Sperm. Theriogenology.

[37] Watanabe Y.F., Souza H.A., Mingoti R.D., Ferreira R.M., Batista E.O.S., Dayan A., Watanabe O.Y., Meirelles F.V., Nogueira M.F.G., Ferraz J.B.S. (2017). Number of Oocytes Retrieved per Donor during OPU and Its Relationship with in Vitro Embryo Production and Field Fertility Following Embryo Transfer. Anim. Reprod..

[38] Batista E., Macedo G., Sala R., Ortolan M., Sá Filho M., Del Valle T., Jesus E., Lopes R., Rennó F., Baruselli P. (2014). Plasma Antimullerian Hormone as a Predictor of Ovarian Antral Follicular Population in *Bos indicus* (Nelore) and *Bos taurus* (Holstein) Heifers. Reprod. Domest. Anim..

[39] Guerreiro B.M., Batista E.O.S., Vieira L.M., Sá Filho M.F., Rodrigues C.A., Castro Netto A., Silveira C.R.A., Bayeux B.M., Dias E.A.R., Monteiro F.M. (2014). Plasma Anti-Mullerian Hormone: An Endocrine Marker for in Vitro Embryo Production from Bos Taurus and Bos Indicus Donors. Domest. Anim. Endocrinol..

[40] Thundathil J., Dance A., Johnson C., Kastelic J. Efficiency of Repeated Superstimulations on Ovum Pick-up and in Vitro Production (OPU-IVP) of Cattle Embryos and Donor Health. Proceedings of the 19th International Congresson Animal Reproduction.

[41] Landry D.A., Bellefleur A.-M., Labrecque R., Grand F.-X., Vigneault C., Blondin P., Sirard M.-A. (2016). Effect of Cow Age on the in Vitro Developmental Competence of Oocytes Obtained after FSH Stimulation and Coasting Treatments. Theriogenology.

[42] Du Y., Xia Y., Xu J., Liu Z., Liu Z., Zhang M., Xu G., Xing X., Du F. (2024). Effects of Donor Age and Reproductive History on Developmental Potential of Ovum Pickup Oocytes in Japanese Black Cattle (Wagyu). Theriogenology.

[43] Khatir H., Lonergan P., Carolan C., Mermillod P. (1996). Prepubertal Bovine Oocyte: A Negative Model for Studying Oocyte Developmental Competence. Mol. Reprod. Dev..

[44] Crowe A.D., Lonergan P., Butler S.T. (2021). Invited Review: Use of Assisted Reproduction Techniques to Accelerate Genetic Gain and Increase Value of Beef Production in Dairy Herds. J. Dairy Sci..

[45] Revel F., Mermillod P., Peynot N., Renard J.P., Heyman Y. (1995). Low Developmental Capacity of in Vitro Matured and Fertilized Oocytes from Calves Compared with That of Cows. Reproduction.

[46] Rizos D., Gutiérrez-Adán A., Pérez-Garnelo S., De La Fuente J., Boland M.P., Lonergan P. (2003). Bovine Embryo Culture in the Presence or Absence of Serum: Implications for Blastocyst Development, Cryotolerance, and Messenger RNA Expression1. Biol. Reprod..

[47] Galli C., Duchi R., Crotti G., Turini P., Ponderato N., Colleoni S., Lagutina I., Lazzari G. (2003). Bovine Embryo Technologies. Theriogenology.

[48] Chaubal S.A., Molina J.A., Ohlrichs C.L., Ferre L.B., Faber D.C., Bols P.E.J., Riesen J.W., Tian X., Yang X. (2006). Comparison of Different Transvaginal Ovum Pick-up Protocols to Optimise Oocyte Retrieval and Embryo Production over a 10-Week Period in Cows. Theriogenology.

[49] Goodhand K.L., Staines M.E., Hutchinson J.S.M., Broadbent P.J. (2000). In Vivo Oocyte Recovery and in Vitro Embryo Production from Bovine Oocyte Donors Treated with Progestagen, Oestradiol and FSH. Anim. Reprod. Sci..

[50] Blondin P., Bousquet D., Twagiramungu H., Barnes F., Sirard M.-A. (2002). Manipulation of Follicular Development to Produce Developmentally Competent Bovine Oocytes1. Biol. Reprod..

[51] Zangirolamo A.F., Morotti F., Silva N.C.D., Sanches T.K., Seneda M.M. (2018). Ovarian Antral Follicle Populations and Embryo Production in Cattle. Anim. Reprod..

[52] Baruselli P.S., Souza A.H.D., Sá M.F.D., Marques M.O., Sales J.N.D.S. (2018). Genetic Market in Cattle (Bull, Ai, Ftai, Moet and Ivp): Financial Payback Based on Reproductive Efficiency in Beef and Dairy Herds in Brazil. Anim. Reprod..

[53] Chasombat J., Nagai T., Parnpai R., Vongpralub T. (2013). Ovarian Follicular Dynamics, Ovarian Follicular Growth, Oocyte Yield, In vitro Embryo Production and Repeated Oocyte Pick Up in Thai Native Heifers Undergoing Superstimulation. Asian-Australas. J. Anim. Sci..

[54] Marshall K.L., Rivera R.M. (2018). The Effects of Superovulation and Reproductive Aging on the Epigenome of the Oocyte and Embryo. Mol. Reprod. Dev..

[55] Durocher J., Morin N., Blondin P. (2006). Effect of Hormonal Stimulation on Bovine Follicular Response and Oocyte Developmental Competence in a Commercial Operation. Theriogenology.

[56] Monteiro F., Ferreira M., Potiens J., Eberhardt B., Trinca L., Barros C. (2009). Influence of Superovulatory Protocols on In Vitro Production of Nellore (*Bos indicus*) Embryos. Reprod. Domest. Anim..

[57] Armstrong D.T., Irvine B.J., Earl C.R., McLean D., Seamark R.F. (1994). Gonadotropin Stimulation Regimens for Follicular Aspiration and in Vitro Embryo Production from Calf Oocytes. Theriogenology.

[58] Currin L., Michalovic L., Bellefleur A.-M., Gutierrez K., Glanzner W., Schuermann Y., Bohrer R.C., Dicks N., Da Rosa P.R., De Cesaro M.P. (2017). The Effect of Age and Length of Gonadotropin Stimulation on the in Vitro Embryo Development of Holstein Calf Oocytes. Theriogenology.

[59] Taneja M., Bols P.E.J., De Velde A.V., Ju J.-C., Schreiber D., Tripp M.W., Levine H., Echelard Y., Riesen J., Yang X. (2000). Developmental Competence of Juvenile Calf Oocytes In Vitro and In Vivo: Influence of Donor Animal Variation and Repeated Gonadotropin Stimulation1. Biol. Reprod..

[60] Viana J.H.M., Silva B.D.M., Moura R.M.D., Féres L.F.R., Figueiredo R.A. (2024). Oocyte Developmental Potential and Embryo Production before Puberty in Cattle. Anim. Reprod..

[61] Kawamoto T.S., Viana J.H.M., Pontelo T.P., Franco M.M., De Faria O.A.C., Fidelis A.A.G., Vargas L.N., Figueiredo R.A. (2022). Dynamics of the Reproductive Changes and Acquisition of Oocyte Competence in Nelore (Bos Taurus Indicus) Calves during the Early and Intermediate Prepubertal Periods. Animals.

[62] Currin L., Baldassarre H., Bordignon V. (2021). In Vitro Production of Embryos from Prepubertal Holstein Cattle and Mediterranean Water Buffalo: Problems, Progress and Potential. Animals.

[63] Michalovic L., Currin L., Gutierrez K., Bellefleur A., Glanzner W.G., Schuermann Y., De Macedo M.P., Bohrer R.C., Dicks N., Lopez R. (2018). Granulosa Cells of Prepubertal Cattle Respond to Gonadotropin Signaling and Upregulate Genes That Promote Follicular Growth and Prevent Cell Apoptosis. Mol. Reprod. Dev..

[64] Nivet A.-L., Bunel A., Labrecque R., Belanger J., Vigneault C., Blondin P., Sirard M.-A. (2012). FSH Withdrawal Improves Developmental Competence of Oocytes in the Bovine Model. Reproduction.

[65] Petrovas G., Kosior M.A., Presicce G.A., Russo M., Zullo G., Albero G., Alkan S., Gasparrini B. (2020). FSH Stimulation with Short Withdrawal Improves Oocyte Competence in Italian Mediterranean Buffalo (*Bubalus bubalis*). Animals.

[66] Oliveira L.H., Sanches C.P., Seddon A.S., Veras M.B., Lima F.A., Monteiro P.L.J., Wiltbank M.C., Sartori R. (2016). Short Communication: Follicle Superstimulation before Ovum Pick-up for in Vitro Embryo Production in Holstein Cows. J. Dairy Sci..

[67] Vieira L.M., Rodrigues C.A., Castro Netto A., Guerreiro B.M., Silveira C.R.A., Moreira R.J.C., Sá Filho M.F., Bó G.A., Mapletoft R.J., Baruselli P.S. (2014). Superstimulation Prior to the Ovum Pick-up to Improve in Vitro Embryo Production in Lactating and Non-Lactating Holstein Cows. Theriogenology.

[68] Chu T., Dufort I., Sirard M.-A. (2012). Effect of Ovarian Stimulation on Oocyte Gene Expression in Cattle. Theriogenology.

[69] Mourot M., Dufort I., Gravel C., Algriany O., Dieleman S., Sirard M. (2006). The Influence of Follicle Size, FSH-enriched Maturation Medium, and Early Cleavage on Bovine Oocyte Maternal mRNA Levels. Mol. Reprod. Dev..

[70] Petyim S., Båge R., Madej A., Larsson B. (2007). Ovum Pick-up in Dairy Heifers: Does It Affect Animal Well-being?. Reprod. Domest. Anim..

[71] Pieterse M.C., Kappen K.A., Kruip T.A.M., Taverne M.A.M. (1988). Aspiration of Bovine Oocytes during Transvaginal Ultrasound Scanning of the Ovaries. Theriogenology.

[72] Kruip T.A.M., Den Daas J.H.G. (1997). In Vitro Produced and Cloned Embryos: Effects on Pregnancy, Parturition and Offspring. Theriogenology.

[73] Dogan H., Yenilmez K. (2024). Factors Influencing Ovum Pick-up Technique Results in Cattle. J. Istanb. Vet. Sci..

[74] Kruip T.A.M., Boni R., Wurth Y.A., Roelofsen M.W.M., Pieterse M.C. (1994). Potential Use of Ovum Pick-up for Embryo Production and Breeding in Cattle. Theriogenology.

[75] Dunning K.R., Robker R.L. (2017). The Role of L-Carnitine during Oocyte in Vitro Maturation: Essential Co-Factor?. Anim. Reprod..

[76] Fischer B., Bavister B.D. (1993). Oxygen Tension in the Oviduct and Uterus of Rhesus Monkeys, Hamsters and Rabbits. Reproduction.

[77] Sciorio R., Smith G.D. (2019). Embryo Culture at a Reduced Oxygen Concentration of 5%: A Mini Review. Zygote.

[78] Steptoe P., Edwards R., Purdy J. (1971). Human Blastocysts Grown in Culture. Nature.

[79] Konstantogianni O., Panou T., Zikopoulos A., Skentou C., Stavros S., Asimakopoulos B. (2024). Culture of Human Embryos at High and Low Oxygen Levels. J. Clin. Med..

[80] Varghese J., Link B., Wong B., Thundathil J.C. (2024). Comparison of the Developmental Competence of in Vitro-Produced Mouse Embryos Cultured under 5 versus 2% O_2_ with in Vivo-Derived Blastocysts. J. Assist. Reprod. Genet..

[81] Rizos D., Ward F., Boland M.P., Lonergan P. (2001). Effect of Culture System on the Yield and Quality of Bovine Blastocysts as Assessed by Survival after Vitrification. Theriogenology.

[82] Gardner D.K., Lane M. (2002). Development of Viable Mammalian Embryos in Vitro. Principles of Cloning.

[83] Thompson J.G., Peterson A.J. (2000). Bovine Embryo Culture in Vitro: New Developments and Post-Transfer Consequences. Hum. Reprod..

[84] Marsico T.V., Camargo J.D., Valente R.S., Sudano M.J. (2019). Embryo Competence and Cryosurvival: Molecular and Cellular Features. Anim. Reprod..

[85] Palasz A.T., Thundathil J., De La Fuente J., Mapletoft R.J. (2000). Effect of Reduced Concentrations of Glycerol and Various Macromolecules on the Cryopreservation of Mouse and Cattle Embryos. Cryobiology.

[86] Edidin M. (2003). Lipids on the Frontier: A Century of Cell-Membrane Bilayers. Nat. Rev. Mol. Cell Biol..

[87] Tharasanit T., Thuwanut P. (2021). Oocyte Cryopreservation in Domestic Animals and Humans: Principles, Techniques and Updated Outcomes. Animals.

[88] Singh B., Mal G., Gautam S.K., Mukesh M. (2019). Advances in Animal Biotechnology.

[89] Choi H.-W., Jang H. (2022). Application of Nanoparticles and Melatonin for Cryopreservation of Gametes and Embryos. Curr. Issues Mol. Biol..

[90] Murray K.A., Gibson M.I. (2022). Chemical Approaches to Cryopreservation. Nat. Rev. Chem..

[91] Sareen S., Talwar P. (2010). Overview of Cryobiology in ART. J. South Asian Fed. Obstet. Gynaecol..

[92] Takahashi T., Inaba Y., Somfai T., Kaneda M., Geshi M., Nagai T., Manabe N. (2013). Supplementation of Culture Medium with L-Carnitine Improves Development and Cryotolerance of Bovine Embryos Produced in Vitro. Reprod. Fertil. Dev..

[93] Lonergan P., Rizos D., Ward F., Boland M.P. (2001). Factors Influencing Oocyte and Embryo Quality in Cattle. Reprod. Nutr. Dev..

[94] Wrenzycki C. (2018). Gene Expression Analysis and in Vitro Production Procedures for Bovine Preimplantation Embryos: Past Highlights, Present Concepts and Future Prospects. Reprod. Domest. Anim..

[95] De Andrade Melo-Sterza F., Poehland R. (2021). Lipid Metabolism in Bovine Oocytes and Early Embryos under in Vivo, in Vitro, and Stress Conditions. Int. J. Mol. Sci..

[96] Keane J.A., Ealy A.D. (2024). An Overview of Reactive Oxygen Species Damage Occurring during In Vitro Bovine Oocyte and Embryo Development and the Efficacy of Antioxidant Use to Limit These Adverse Effects. Animals.

[97] Di Meo S., Reed T.T., Venditti P., Victor V.M. (2016). Role of ROS and RNS Sources in Physiological and Pathological Conditions. Oxid. Med. Cell. Longev..

[98] Silva B.R., Silva J.R.V. (2023). Mechanisms of Action of Non-Enzymatic Antioxidants to Control Oxidative Stress during in Vitro Follicle Growth, Oocyte Maturation, and Embryo Development. Anim. Reprod. Sci..

[99] Gutiérrez-Añez J.C., Henning H., Lucas-Hahn A., Baulain U., Aldag P., Sieg B., Hensel V., Herrmann D., Niemann H. (2021). Melatonin Improves Rate of Monospermic Fertilization and Early Embryo Development in a Bovine IVF System. PLoS ONE.

[100] Salzano A., Albero G., Zullo G., Neglia G., Abdel-Wahab A., Bifulco G., Zicarelli L., Gasparrini B. (2014). Effect of Resveratrol Supplementation during Culture on the Quality and Cryotolerance of Bovine in Vitro Produced Embryos. Anim. Reprod. Sci..

[101] Soto-Heras S., Paramio M.-T. (2020). Impact of Oxidative Stress on Oocyte Competence for in Vitro Embryo Production Programs. Res. Vet. Sci..

[102] Di Emidio G., Rea F., Placidi M., Rossi G., Cocciolone D., Virmani A., Macchiarelli G., Palmerini M.G., D’Alessandro A.M., Artini P.G. (2020). Regulatory Functions of L-Carnitine, Acetyl, and Propionyl L-Carnitine in a PCOS Mouse Model: Focus on Antioxidant/Antiglycative Molecular Pathways in the Ovarian Microenvironment. Antioxidants.

[103] Lin T., Lee J.E., Kang J.W., Oqani R.K., Cho E.S., Kim S.B., Il Jin D. (2018). Melatonin Supplementation during Prolonged in Vitro Maturation Improves the Quality and Development of Poor-quality Porcine Oocytes via Anti-oxidative and Anti-apoptotic Effects. Mol. Reprod. Dev..

[104] Pero M.E., Zullo G., Esposito L., Iannuzzi A., Lombardi P., De Canditiis C., Neglia G., Gasparrini B. (2018). Inhibition of Apoptosis by Caspase Inhibitor Z-VAD-FMK Improves Cryotolerance of in Vitro Derived Bovine Embryos. Theriogenology.

[105] Dias L.R.O., Leme L.O., Sprícigo J.F.W., Pivato I., Dode M.A.N. (2020). Effect of Delipidant Agents during in Vitro Culture on the Development, Lipid Content, Gene Expression and Cryotolerance of Bovine Embryos. Reprod. Domest. Anim..

[106] Aizawa R., Ibayashi M., Tatsumi T., Yamamoto A., Kokubo T., Miyasaka N., Sato K., Ikeda S., Minami N., Tsukamoto S. (2019). Synthesis and Maintenance of Lipid Droplets Are Essential for Mouse Preimplantation Embryonic Development. Development.

[107] Brown D.A. (2001). Lipid Droplets: Proteins Floating on a Pool of Fat. Curr. Biol..

[108] Paczkowski M., Silva E., Schoolcraft W.B., Krisher R.L. (2013). Comparative Importance of Fatty Acid Beta-Oxidation to Nuclear Maturation, Gene Expression, and Glucose Metabolism in Mouse, Bovine, and Porcine Cumulus Oocyte Complexes. Biol. Reprod..

[109] Sturmey R., Reis A., Leese H., McEvoy T. (2009). Role of Fatty Acids in Energy Provision During Oocyte Maturation and Early Embryo Development. Reprod. Domest. Anim..

[110] Kajdasz A., Warzych E., Derebecka N., Madeja Z.E., Lechniak D., Wesoly J., Pawlak P. (2020). Lipid Stores and Lipid Metabolism Associated Gene Expression in Porcine and Bovine Parthenogenetic Embryos Revealed by Fluorescent Staining and RNA-Seq. Int. J. Mol. Sci..

[111] Sastre D., Costa N.N.D., Sá A.L.A.D., Conceição S.D.B., Chiaratti M.R., Adona P.R., Guemra S., Meirelles F.V., Santos S.D.S.D., Sena L. (2014). Expression of PLIN2 and PLIN3 during Oocyte Maturation and Early Embryo Development in Cattle. Theriogenology.

[112] Schwarz K.R.L., De Castro F.C., Schefer L., Botigelli R.C., Paschoal D.M., Fernandes H., Leal C.L.V. (2018). The Role of cGMP as a Mediator of Lipolysis in Bovine Oocytes and Its Effects on Embryo Development and Cryopreservation. PLoS ONE.

[113] Prates E.G., Nunes J.T., Pereira R.M. (2014). A Role of Lipid Metabolism during Cumulus-Oocyte Complex Maturation: Impact of Lipid Modulators to Improve Embryo Production. Mediators Inflamm..

[114] Shi M., Sirard M.-A. (2022). Metabolism of Fatty Acids in Follicular Cells, Oocytes, and Blastocysts. Reprod. Fertil..

[115] Janati Idrissi S., Le Bourhis D., Lefevre A., Emond P., Le Berre L., Desnoës O., Joly T., Buff S., Maillard V., Schibler L. (2021). Lipid Profile of Bovine Grade-1 Blastocysts Produced Either in Vivo or in Vitro before and after Slow Freezing Process. Sci. Rep..

[116] Arroyave-Ospina J.C., Wu Z., Geng Y., Moshage H. (2021). Role of Oxidative Stress in the Pathogenesis of Non-Alcoholic Fatty Liver Disease: Implications for Prevention and Therapy. Antioxidants.

[117] Jomova K., Raptova R., Alomar S.Y., Alwasel S.H., Nepovimova E., Kuca K., Valko M. (2023). Reactive Oxygen Species, Toxicity, Oxidative Stress, and Antioxidants: Chronic Diseases and Aging. Arch. Toxicol..

[118] Del Collado M., Saraiva N.Z., Lopes F.L., Gaspar R.C., Padilha L.C., Costa R.R., Rossi G.F., Vantini R., Garcia J.M. (2016). Influence of Bovine Serum Albumin and Fetal Bovine Serum Supplementation during in Vitro Maturation on Lipid and Mitochondrial Behaviour in Oocytes and Lipid Accumulation in Bovine Embryos. Reprod. Fertil. Dev..

[119] Koch E., Hopmann C., Fröhlich L., Schebb N.H. (2021). Fatty Acid and Oxylipin Concentration Differ Markedly between Different Fetal Bovine Serums: A Cautionary Note. Lipids.

[120] Reis A., Rooke J.A., McCallum G.J., Staines M.E., Ewen M., Lomax M.A., McEvoy T.G. (2003). Consequences of Exposure to Serum, with or without Vitamin E Supplementation, in Terms of the Fatty Acid Content and Viability of Bovine Blastocysts Produced in Vitro. Reprod. Fertil. Dev..

[121] Nonogaki T., Noda Y., Goto Y., Kishi J., Mori T. (1994). Developmental Blockage of Mouse Embryos Caused by Fatty Acids. J. Assist. Reprod. Genet..

[122] Mostafa S., Nader N., Machaca K. (2022). Lipid Signaling During Gamete Maturation. Front. Cell Dev. Biol..

[123] Kim J.Y., Kinoshita M., Ohnishi M., Fukui Y. (2001). Lipid and Fatty Acid Analysis of Fresh and Frozen–Thawed Immature and in Vitro Matured Bovine Oocytes. Reproduction.

[124] Zeron Y., Sklan D., Arav A. (2002). Effect of Polyunsaturated Fatty Acid Supplementation on Biophysical Parameters and Chilling Sensitivity of Ewe Oocytes. Mol. Reprod. Dev..

[125] Chen P.R., Redel B.K., Kerns K.C., Spate L.D., Prather R.S. (2021). Challenges and Considerations during In Vitro Production of Porcine Embryos. Cells.

[126] Nagashima H., Kashiwazaki N., Ashman R.J., Grupen C.G., Seamark R.F., Nottle M.B. (1994). Removal of Cytoplasmic Lipid Enhances the Tolerance of Porcine Embryos to Chilling. Biol. Reprod..

[127] Li R., Murphy C.N., Spate L., Wax D., Isom C., Rieke A., Walters E.M., Samuel M., Prather R.S. (2009). Production of Piglets after Cryopreservation of Embryos Using a Centrifugation-Based Method for Delipation Without Micromanipulation1. Biol. Reprod..

[128] Pereira R.M., Marques C.C. (2008). Animal Oocyte and Embryo Cryopreservation. Cell Tissue Bank..

[129] Panyaboriban S., Tharasanit T., Chankitisakul V., Swangchan-Uthai T., Techakumphu M. (2018). Treatment with Chemical Delipidation Forskolin Prior to Cryopreservation Improves the Survival Rates of Swamp Buffalo (*Bubalus bubalis*) and Bovine (*Bos indicus*) in Vitro Produced Embryos. Cryobiology.

[130] Leal C.L.V., Cañón-Beltrán K., Cajas Y.N., Hamdi M., Yaryes A., Millán De La Blanca M.G., Beltrán-Breña P., Mazzarella R., Da Silveira J.C., Gutiérrez-Adán A. (2022). Extracellular Vesicles from Oviductal and Uterine Fluids Supplementation in Sequential in Vitro Culture Improves Bovine Embryo Quality. J. Anim. Sci. Biotechnol..

[131] Mazzarella R., Cañón-Beltrán K., Cajas Y.N., Hamdi M., González E.M., Da Silveira J.C., Leal C.L.V., Rizos D. (2024). Extracellular Vesicles-Coupled miRNAs from Oviduct and Uterus Modulate Signaling Pathways Related to Lipid Metabolism and Bovine Early Embryo Development. J. Anim. Sci. Biotechnol..

[132] Lonergan P., Rizos D., Gutierrez-Adan A., Fair T., Boland M. (2003). Oocyte and Embryo Quality: Effect of Origin, Culture Conditions and Gene Expression Patterns. Reprod. Domest. Anim..

[133] Li N., Zhao H. (2021). Role of Carnitine in Non-Alcoholic Fatty Liver Disease and Other Related Diseases: An Update. Front. Med..

[134] Sharma B., Schmidt L., Nguyen C., Kiernan S., Dexter-Meldrum J., Kuschner Z., Ellis S., Bhatia N.D., Agriantonis G., Whittington J. (2024). The Effect of L-Carnitine on Critical Illnesses Such as Traumatic Brain Injury (TBI), Acute Kidney Injury (AKI), and Hyperammonemia (HA). Metabolites.

[135] Rebouche C.J. (2004). Kinetics, Pharmacokinetics, and Regulation of l-Carnitine and Acetyl-l-carnitine Metabolism. Ann. N. Y. Acad. Sci..

[136] Agarwal A., Sengupta P., Durairajanayagam D. (2018). Role of L-Carnitine in Female Infertility. Reprod. Biol. Endocrinol..

[137] Montjean D., Entezami F., Lichtblau I., Belloc S., Gurgan T., Menezo Y. (2012). Carnitine Content in the Follicular Fluid and Expression of the Enzymes Involved in Beta Oxidation in Oocytes and Cumulus Cells. J. Assist. Reprod. Genet..

[138] Placidi M., Di Emidio G., Virmani A., D’Alfonso A., Artini P.G., D’Alessandro A.M., Tatone C. (2022). Carnitines as Mitochondrial Modulators of Oocyte and Embryo Bioenergetics. Antioxidants.

[139] Liang Y., Yoisungnern T., Huang Y., Parnpai R. (2020). Effects of L-Carnitine on Embryo Development of Vitrified Swamp Buffalo Oocytes Following in Vitro Fertilization. Livest. Sci..

[140] Dunning K.R., Akison L.K., Russell D.L., Norman R.J., Robker R.L. (2011). Increased Beta-Oxidation and Improved Oocyte Developmental Competence in Response to L-Carnitine During Ovarian In Vitro Follicle Development in Mice. Biol. Reprod..

[141] Yamada T., Imai H., Yamada M. (2006). Beneficial effects of acetyl-L-carnitine treatment during IVM on post-fertilization development of bovine oocytes in vitro. Reprod. Fertil. Dev..

[142] Arduini A., Dottori S., Sciarroni A.F., Corsico N., Morabito E., Arrigoni-Martelli E., Calvani M. (1995). Effect of Propionyl-L-Carnitine Treatment on Membrane Phospholipid Fatty Acid Turnover in Diabetic Rat Erythrocytes. Mol. Cell. Biochem..

[143] Arenas J., Rubio J.C., Martín M.A., Campos Y. (1998). Biological Roles of L-Carnitine in Perinatal Metabolism. Early Hum. Dev..

[144] Li J., Liu L., Weng J., Yin T., Yang J., Feng H.L. (2021). Biological Roles of L-Carnitine in Oocyte and Early Embryo Development. Mol. Reprod. Dev..

[145] Arduini A., Denisova N., Virmani A., Avrova N., Federici G., Arrigoni-Martelli E. (1994). Evidence for the Involvement of Carnitine-Dependent Long-Chain Acyltransferases in Neuronal Triglyceride and Phospholipid Fatty Acid Turnover. J. Neurochem..

[146] Xu H.-Y., Geng S.-S., Li T.-T., Fu Q., Lu S.-S., Liang X.-W., Lu Y.-Q., Zhang M., Yang X.-G., Lu K.-H. (2019). Maturation of Buffalo Oocytes in Vitro with Acetyl-L-Carnitine Improves Cryotolerance Due to Changes in Mitochondrial Function and the Membrane Lipid Profile. Reprod. Fertil. Dev..

[147] Nagao B., Kobayashi A., Yamazaki N. (1987). Effects of L-Carnitine on Phospholipids in the Ischemic Myocardium. Jpn. Heart J..

[148] Ghanem N., Fakruzzaman M., Batawi A.H., Kong I.-K. (2022). Post-Thaw Viability, Developmental and Molecular Deviations in in Vitro Produced Bovine Embryos Cultured with l-Carnitine at Different Levels of Fetal Calf Serum. Theriogenology.

[149] Sharma J., Antenos M., Madan P. (2021). A Comparative Analysis of Hippo Signaling Pathway Components during Murine and Bovine Early Mammalian Embryogenesis. Genes.

[150] Sonnen K.F., Janda C.Y. (2021). Signalling Dynamics in Embryonic Development. Biochem. J..

[151] Yu F.-X., Zhao B., Panupinthu N., Jewell J.L., Lian I., Wang L.H., Zhao J., Yuan H., Tumaneng K., Li H. (2012). Regulation of the Hippo-YAP Pathway by G-Protein-Coupled Receptor Signaling. Cell.

[152] Rausch V., Hansen C.G. (2020). The Hippo Pathway, YAP/TAZ, and the Plasma Membrane. Trends Cell Biol..

[153] Liu X., Wang Y., Chen B., Chan W.N., Mui C.W., Cheung A.H.K., Zhang J., Wong K.Y., Yu J., Kang W. (2022). Targeting the Hippo Pathway in Gastric Cancer and Other Malignancies in the Digestive System: From Bench to Bedside. Biomedicines.

[154] Negrón-Pérez V.M., Zhang Y., Hansen P.J. (2017). Single-Cell Gene Expression of the Bovine Blastocyst. Reproduction.

[155] Sharma J., Madan P. (2022). Differential Regulation of Hippo Signaling Pathway Components between 8-cell and Blastocyst Stages of Bovine Preimplantation Embryogenesis. Mol. Reprod. Dev..

[156] Hansen C.G., Moroishi T., Guan K.-L. (2015). YAP and TAZ: A Nexus for Hippo Signaling and Beyond. Trends Cell Biol..

[157] Yu B., Van Tol H.T.A., Oei C.H.Y., Stout T.A.E., Roelen B.A.J. (2021). Lysophosphatidic Acid Accelerates Bovine In Vitro-Produced Blastocyst Formation through the Hippo/YAP Pathway. Int. J. Mol. Sci..

[158] Han H., Qi R., Zhou J.J., Ta A.P., Yang B., Nakaoka H.J., Seo G., Guan K.-L., Luo R., Wang W. (2018). Regulation of the Hippo Pathway by Phosphatidic Acid-Mediated Lipid-Protein Interaction. Mol. Cell.

[159] Mo J.-S., Meng Z., Kim Y.C., Park H.W., Hansen C.G., Kim S., Lim D.-S., Guan K.-L. (2015). Cellular Energy Stress Induces AMPK-Mediated Regulation of YAP and the Hippo Pathway. Nat. Cell Biol..

[160] DeRan M., Yang J., Shen C.-H., Peters E.C., Fitamant J., Chan P., Hsieh M., Zhu S., Asara J.M., Zheng B. (2014). Energy Stress Regulates Hippo-YAP Signaling Involving AMPK-Mediated Regulation of Angiomotin-like 1 Protein. Cell Rep..

[161] Santinon G., Pocaterra A., Dupont S. (2016). Control of YAP/TAZ Activity by Metabolic and Nutrient-Sensing Pathways. Trends Cell Biol..

[162] Foretz M., Even P.C., Viollet B. (2018). AMPK Activation Reduces Hepatic Lipid Content by Increasing Fat Oxidation In Vivo. Int. J. Mol. Sci..

[163] Shahouzehi B., Fallah H., Masoumi-Ardakani Y.I. (2020). L-Carnitine Administration Effects on AMPK, APPL1 and PPAR? Genes Expression in the Liver and Serum Adiponectin Levels and HOMA-IR in Type 2 Diabetes Rat Model Induced by STZ and Nicotinamide. Ukr. Biochem. J..

[164] Zhang Y., Fu Y., Jiang T., Liu B., Sun H., Zhang Y., Fan B., Li X., Qin X., Zheng Q. (2021). Enhancing Fatty Acids Oxidation via L-Carnitine Attenuates Obesity-Related Atrial Fibrillation and Structural Remodeling by Activating AMPK Signaling and Alleviating Cardiac Lipotoxicity. Front. Pharmacol..

[165] Sue Y.-M., Chou H.-C., Chang C.-C., Yang N.-J., Chou Y., Juan S.-H. (2014). L-Carnitine Protects against Carboplatin-Mediated Renal Injury: AMPK- and PPARα-Dependent Inactivation of NFAT3. PLoS ONE.

[166] Clark K.L., George J.W., Przygrodzka E., Plewes M.R., Hua G., Wang C., Davis J.S. (2022). Hippo Signaling in the Ovary: Emerging Roles in Development, Fertility, and Disease. Endocr. Rev..

[167] Sorrentino G., Ruggeri N., Specchia V., Cordenonsi M., Mano M., Dupont S., Manfrin A., Ingallina E., Sommaggio R., Piazza S. (2014). Metabolic Control of YAP and TAZ by the Mevalonate Pathway. Nat. Cell Biol..

[168] Guerra B., Recio C., Aranda-Tavío H., Guerra-Rodríguez M., García-Castellano J.M., Fernández-Pérez L. (2021). The Mevalonate Pathway, a Metabolic Target in Cancer Therapy. Front. Oncol..

[169] Wang Z., Wu Y., Wang H., Zhang Y., Mei L., Fang X., Zhang X., Zhang F., Chen H., Liu Y. (2014). Interplay of Mevalonate and Hippo Pathways Regulates RHAMM Transcription via YAP to Modulate Breast Cancer Cell Motility. Proc. Natl. Acad. Sci. USA.

[170] Ortega Á., Vera I., Diaz M., Navarro C., Rojas M., Torres W., Parra H., Salazar J., De Sanctis J., Bermúdez V. (2021). The YAP/TAZ Signaling Pathway in the Tumor Microenvironment and Carcinogenesis: Current Knowledge and Therapeutic Promises. Int. J. Mol. Sci..

[171] Kashiwagi A., Kanno T., Arita K., Ishisaka R., Utsumi T., Utsumi K. (2001). Suppression of T3- and Fatty Acid-Induced Membrane Permeability Transition by l-Carnitine. Comp. Biochem. Physiol. B Biochem. Mol. Biol..

